# Association between epicardial adipose tissue, high-sensitivity C-reactive protein and myocardial dysfunction in middle-aged men with suspected metabolic syndrome

**DOI:** 10.1186/s12933-018-0735-7

**Published:** 2018-06-30

**Authors:** Dong-Hyuk Cho, Hyung Joon Joo, Mi-Na Kim, Do-Sun Lim, Wan Joo Shim, Seong-Mi Park

**Affiliations:** Division of Cardiology, Korea University College of Medicine, Anam Hospital, Inchonro 73, Seongbukgu, Seoul, 136-705 Republic of Korea

**Keywords:** Epicardial adipose tissue, Sex, Global longitudinal strain, High-sensitivity C-reactive protein, Metabolic syndrome

## Abstract

**Background:**

As body fat composition and metabolism differ between men and women, we evaluated sex-related differences in the association among epicardial adipose tissue (EAT), secretome profile, and myocardial function of subjects with suspected metabolic syndrome.

**Methods:**

We evaluated 277 participants (men, n = 140; 56.1 ± 4.7 years) who underwent conventional echocardiography and two-dimensional speckle tracking from the Seoul Metabolic Syndrome cohort. EAT was measured from the right ventricular free wall perpendicular to the aortic annulus at end systole. Global longitudinal strain (GLS) was obtained from 18 apical segments. Apolipoprotein A1, apolipoprotein B, adiponectin, and high-sensitivity C-reactive protein (hs-CRP) levels were measured using immunoturbidimetry assay.

**Results:**

Mean age, body mass index, and hs-CRP level did not differ by sex. Waist circumference, fasting blood glucose level, and triglyceride/high-density lipoprotein cholesterol ratio were higher, and apolipoprotein AI and adiponectin levels were lower in men. No significant difference in mean EAT thickness was found (7.02 ± 1.81 vs. 7.13 ± 1.70 mm, p = 0.613). Men had a higher left ventricular (LV) mass index and lower GLS. EAT thickness was associated with hs-CRP level in men alone (*ß *= 0.206, p = 0.015). LV mass index (*ß *= 2.311, p = 0.037) and function represented by *e*′ (*ß *= − 0.279, p = 0.001) and GLS (*ß *= − 0.332, p < 0.001) were independently associated with EAT thickness in men alone.

**Conclusions:**

In middle-aged subjects with suspected metabolic syndrome, EAT was associated with inflammation represented by hs-CRP level, LV mass, and subclinical myocardial dysfunction only in men, suggesting that the inflammatory activity of EAT induced myocardial remodeling and dysfunction in middle-aged subjects but was attenuated in women.

*Trial registration* NCT02077530 (date of registration: November 1, 2013)

## Introduction

Middle-aged men are known to be at higher risk than women for cardiovascular diseases (CVD) [1, 2]. Previous studies report that higher metabolic risk factors in men and the protective effect of estrogen in premenopausal women may contribute to the sex-related differences in the middle-aged population [1, 3, 4]. However, the underlying mechanisms of the observed sex-related differences are still unclear.

Generally, women have more body fat than men with a similar body mass index (BMI) [5]. Body fat composition and metabolism differ between men and women [6–8]. Men tend to have more visceral adipose tissue (VAT), whereas women tend to have more subcutaneous adipose tissue [9]. Sex hormones, including estrogen and testosterone, appear to play a significant role in these differences [7].

Epicardial adipose tissue (EAT) is an easily measurable VAT by echocardiography [10]. EAT is metabolically bioactive and the source of adipocytokines, proatherogenic mediators, and pro-inflammatory cytokines [11–15]. EAT may have important roles in the pathophysiologies of metabolic syndrome and cardiometabolic diseases [16–19]. The association of EAT accumulation with subclinical changes in myocardial structure and function has been reported [20–22]. Although several studies reported that EAT increases in women after menopause and the relationship between EAT thickness and myocardial function are significant only in women after 60 years old [21, 23], the sex-related difference in EAT thickness and its association with myocardial function have not been investigated in the middle-aged population.

Therefore, we investigated the association of EAT thickness with adipocytokine levels, inflammatory marker level, and myocardial function, and the sex-related difference in its association in a middle-aged population.

## Methods

### Study population

The Seoul Metabolic Syndrome study is a prospective ongoing cohort study that aims to evaluate the clinical characteristics and outcomes of Koreans with suspected metabolic syndrome. Detailed protocols were reported in previously published papers [24, 25]. Participants from 30 to 64 years of age were enrolled from 25 public healthcare centers between January 2014 and September 2014. Participants with a previous history of stroke, angina pectoris, myocardial infarction, or any revascularization were excluded from the enrollment. Demographic data, medical history of traditional cardiovascular risk factors, and medications were assessed through a standardized questionnaire. Basic physical examinations were performed by physicians. Among 1130 participants in the cohort, 277 who underwent both conventional echocardiography and two-dimensional (2-D) speckle tracking were enrolled in this study. This study protocol was approved by the institutional review board of the Korea University Anam Hospital, and written informed consent was obtained from each participant (IRB NO. ED13087).

### Anthropometric and laboratory parameters

Body weight, height, waist and hip circumferences, and systolic (SBP) and diastolic blood pressures (DBP) were measured on the day of medical examination. All the subjects fasted for at least 8 h before blood sampling at the first visit. Fasting blood glucose (FBG) level was measured using an ultraviolet assay (Roche, Germany). Levels of total cholesterol (TC), triglycerides, low-density lipoprotein (LDL)-cholesterol, and high-density lipoprotein (HDL)-cholesterol were determined using the homogeneous enzymatic colorimetric assay (Roche, Germany). Apolipoprotein A1, apolipoprotein B, and high-sensitivity C-reactive protein (hs-CRP) levels were measured using immunoturbidimetry assay (Roche, Germany). Adiponectin was measured by using multiplex immunoassays (Millipore, USA). All laboratory analyses were performed by Green Cross Laboratories (Gyunggi, Korea). Each assay was controlled within 5% value of coefficient of variation every day.

### Conventional echocardiography

Two-dimensional/Doppler echocardiography was performed in each subject by using a commercially available echocardiographic system (Vivid-E9, Vingmed-General Electric, Horten, Norway) with a M5Sc transducer. Chamber quantification was performed from 2-D echocardiography images. Left atrial (LA) volume index (LAVI) and left ventricular (LV) mass index (LVMI) were calculated using the formula recommended by the American Society of Echocardiography [26]. Mitral inflow velocity was obtained in the apical four-chamber view by pulsed-wave Doppler echocardiography during early (E) and late filling (A). The early diastolic mitral annular velocity (*e*′) of the septal mitral and lateral mitral annuli were evaluated using tissue Doppler imaging. LV global longitudinal strain (GLS) was obtained from 18 apical segments and analyzed using the ECHOPAC PC software (version 201.67.0).

### EAT thickness measurement

EAT was defined as the relatively echo-free space between the outer wall of the myocardium and the visceral layer of the pericardium [10]. The parasternal long-axis view was performed to measure the maximal EAT thickness from the right ventricular free wall perpendicular to the aortic annulus at end systole. The values were measured in three cardiac cycles and were averaged. For the reliability of the EAT thickness measurement, the interclass correlation coefficients for the intraobserver and interobserver variability were 0.992 (95% confidence interval [CI] 0.983–0.996; p < 0.001) and 0.902 (95% CI 0.795–0.953, p < 0.001), respectively.

### Statistical analyses

All results were presented as mean ± SD for continuous variables or as frequencies (percentages) for categorical variables. The differences in the frequency of cardiovascular risk factors between the men and women were compared using the Chi square test, and the differences in the EAT thickness and echocardiographic parameters were compared using the Student *t* test. The correlations among EAT thickness, cardiovascular risk factors, and laboratory and echocardiographic parameters were evaluated using the correlation analysis. The mean hs-CRP level was compared according to the median value of myocardial dysfunction by Student *t* test. Multiple linear regression analysis was performed to access the association between EAT thickness and myocardial function after adjustment for age, BMI, systolic blood pressure, fasting glucose level, triglyceride level, high-density lipoprotein level and LVMI. SPSS version 22.0 for Windows (IBM, NY, USA) was used to perform the statistical analysis. A two-sided p value of < 0.05 was considered statistically significant.

## Results

### Baseline characteristics

In this study, 277 participants (140 men and 137 women, 56.1 ± 4.71 years) were enrolled. The baseline characteristics of the men and women are shown in Table 1. Mean age and BMI did not differ between the men and women; however, waist circumference, SBP, and DBP were greater in the men than in the women. No significant differences in the prevalence of hypertension, diabetes mellitus, and dyslipidemia were found between the sexes.

**Table 1:** Baseline characteristics of men and women, including laboratory and echocardiographic parameters

	Men	Women	p
Anthropometric parameters			
Age (years)	56.3 ± 5.0	55.9 ± 4.4	0.561
Body mass index (kg/m^2^)	25.9 ± 2.8	25.5 ± 2.9	0.207
Waist (cm)	90.6 ± 6.6	84.5 ± 7.6	< 0.001
Systolic blood pressure (mm Hg)	126.5 ± 14.4	121.2 ± 15.1	0.003
Diastolic blood pressure (mm Hg)	78.1 ± 9.8	74.2 ± 9.1	0.001
Underlying diseases			
Hypertension (n [%])	12 (9.1%)	6 (4.6%)	0.302
Diabetes mellitus (n [%])	3 (2.3%)	1 (0.8%)	0.513
Dyslipidemia (n [%])	16 (12.1%)	7 (5.3%)	0.079
Laboratory findings			
Fasting blood glucose (mg/dl)	104.0 ± 16.2	95.8 ± 11.5	< 0.001
Total cholesterol (mg/dl)	192.4 ± 32.3	204.3 ± 31.7	0.002
LDL-cholesterol (mg/dl)	123.5 ± 32.6	134.0 ± 31.9	0.007
Triglyceride (mg/dl)	170.8 ± 105.0	138.3 ± 77.4	0.004
HDL-cholesterol (mg/dl)	48.3 ± 13.5	54.3 ± 13.0	< 0.001
TG/HDL ratio	4.1 ± 4.0	2.9 ± 2.4	0.003
Apolipoprotein AI (mg/dl)	138.8 ± 26.5	145.8 ± 24.1	0.02
Apolipoprotein B (mg/dl)	100.8 ± 21.6	101.8 ± 24.1	0.722
Adiponectin (ng/ml)	7.5 ± 4.9	12.4 ± 6.3	< 0.001
WBC count (× 10^3^/μl)	3.93 ± 2.70	3.96 ± 3.10	0.937
High-sensitivity CRP (mg/dl)	1.39 ± 2.58	1.21 ± 1.74	0.513
Echocardiographic parameters			
EAT thickness (mm)	7.02 ± 1.81	7.13 ± 1.70	0.613
LVMI (g/m^2^)	75.0 ± 23.2	68.6 ± 14.3	0.006
LAVI (ml/m^2^)	24.2 ± 8.5	26.8 ± 7.1	0.006
EF (%)	58.5 ± 6.4	59.4 ± 9.1	0.352
E (cm/s)	51.5 ± 12.6	56.3 ± 13.1	0.002
A (cm/s)	55.7 ± 16.7	59.9 ± 14.7	0.029
E/A ratio	1.0 ± 0.8	1.0 ± 0.3	0.516
DT (ms)	193.5 ± 43.6	187.6 ± 41.9	0.263
*e*′ (cm/s)	6.3 ± 1.5	6.3 ± 1.6	0.839
*E*/*e*′	7.0 ± 1.8	7.8 ± 1.9	< 0.001
GLS (%)	− 15.3 ± 2.3	− 17.0 ± 2.0	< 0.001

Data are presented as number (%) for categorical variables or mean ± standard deviation for continuous variables

LDL, low-density lipoprotein; HDL, high-density lipoprotein; TG/HDL, triglyceride/HDL-cholesterol; WBC, white blood cell; CRP, C-reactive protein; LVMI, left ventricular mass index; LAVI, left atrial volume index; EF, ejection fraction; *E*, early diastolic mitral inflow velocity; DT, deceleration time; *e*′, septal early mitral tissue velocity; *a*′, septal late mitral tissue velocity; *s*′, septal systolic mitral tissue velocity; GLS, global longitudinal strain

Metabolic laboratory parameters represented by FBG, LDL, TG, HDL, apolipoprotein AI, and adiponectin levels were more impaired in the men than in the women. However, hs-CRP level as a systemic inflammatory marker and apolipoprotein B level were not different between the men and women.

LVMI was higher in the men, but LAVI was higher in the women. Ejection fraction and septal *e*′ velocity did not differ between the sexes. GLS was more impaired in the men. The mean EAT thickness was 7.07 ± 1.76 mm, without significant difference between the men and women.

### EAT and its characteristics in all the participants

EAT thickness significantly correlated with obesity represented by BMI (*r* = 0.286, p < 0.001) and waist circumference (*r* = 0.321, p < 0.001). Among the lipid profiles, including apolipoprotein and adiponectin levels, only TG level was weakly associated with EAT thickness (*r* = 0.121, p = 0.044). An inflammatory marker assessed using hs-CRP level was significantly associated with EAT thickness (*r* = 0.176, p = 0.003). LVMI (*r* = 0.128, p = 0.034), GLS (*r* = − 0.235, p < 0.001), and LV diastolic function represented by septal *e*′ velocity (*r* = − 0.205, p = 0.001) and *E*/*e*′ ratio (*r* = 0.137, p = 0.023) also significantly correlated with EAT thickness.

### Systemic inflammation according to sex and myocardial dysfunction

**Fig. 1:**
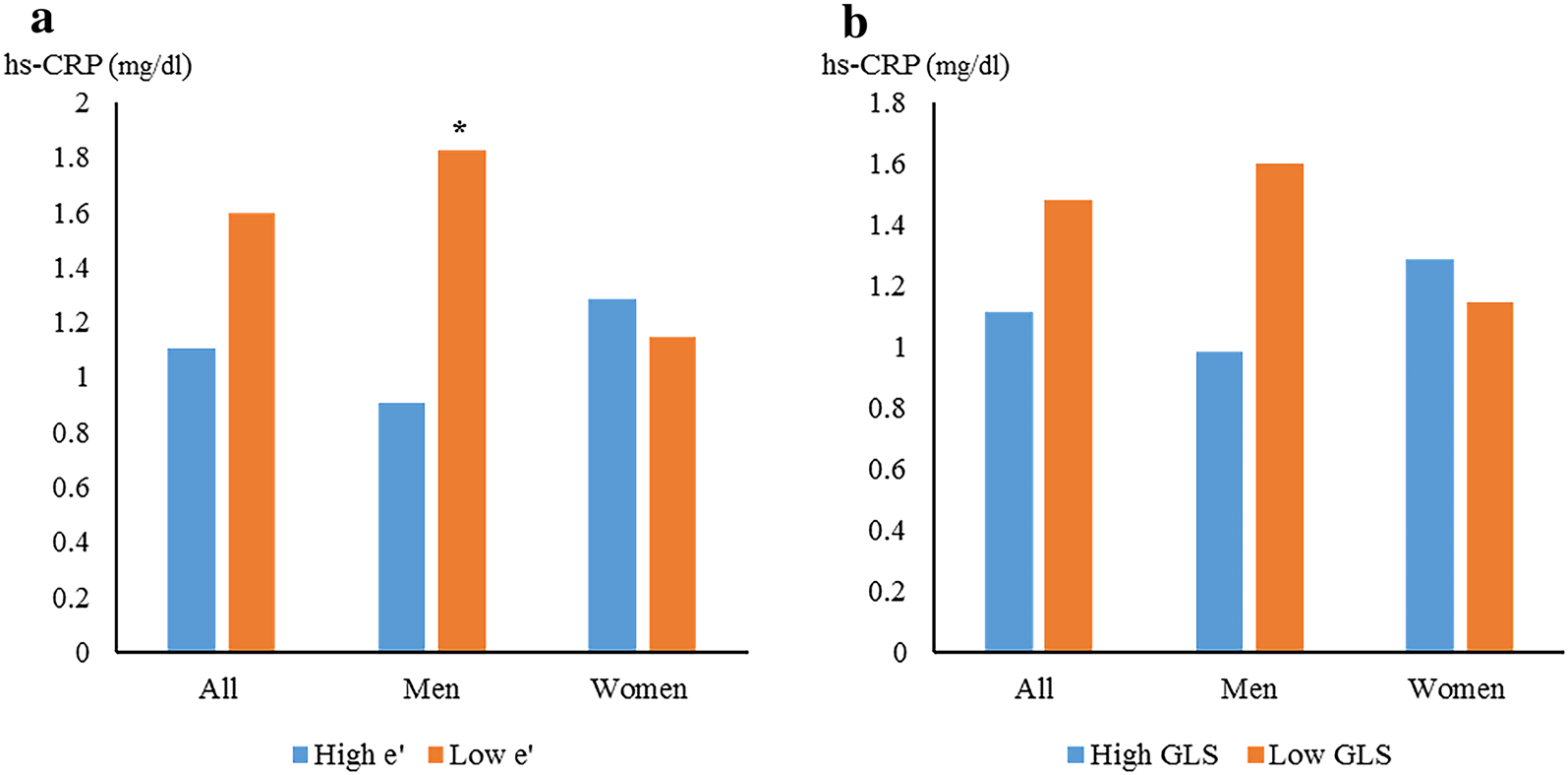
Comparison of high-sensitivity C-reactive protein levels according to median *e*′ velocity (**a**), and GLS (**b**). * means p-value is less than 0.05 compared with the high-sensitivity C-reactive protein level of the subjects without myocardial dysfunction in each group

To investigate the association between systemic inflammation and myocardial function, the mean hs-CRP level was compared between the men and women according to the medial value of *e*′ velocity and GLS. Only the men demonstrated a higher mean hs-CRP level in the low septal *e*′ velocity group (Fig. 1a). The men with low GLS had lower hs-CRP levels than the men with high GLS, but the difference was not statistically significant (Fig. 1b).

### Sex-related difference in EAT in association with secretome profile and myocardial function

**Fig. 2:**
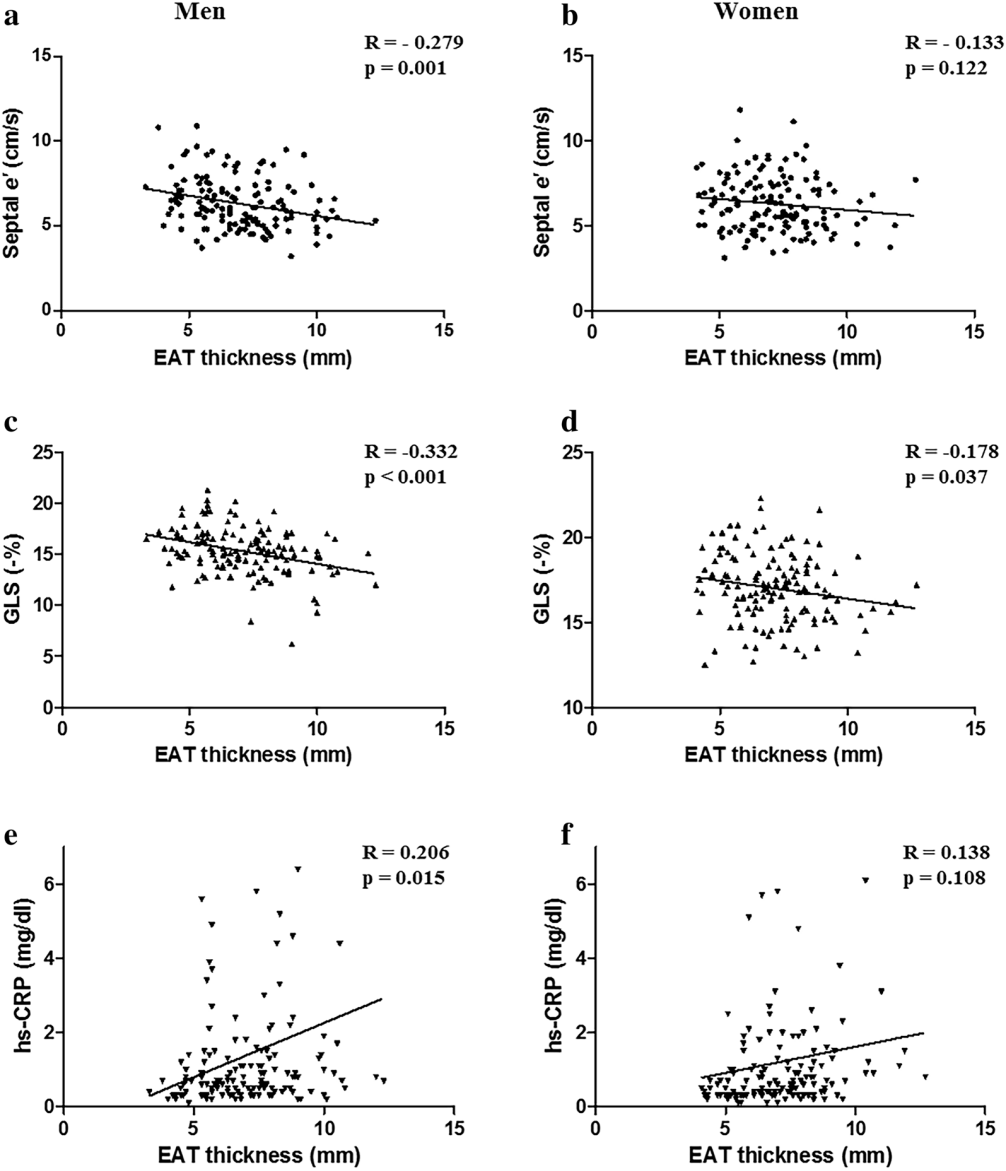
The association of epicardial adipose tissue thickness with myocardial function (**a**–**d**) and high-sensitivity C-reactive protein level (**e**, **f**) in men and women

LVMI, septal *e*′ velocity, and hs-CRP, which were related to EAT thickness in all subjects, were significantly correlated with EAT thickness exclusively in men. GLS was associated with EAT thickness in both the men and women (Fig. 2). The association between EAT thickness and myocardial dysfunction was evaluated using multiple linear regression analysis. EAT thickness was independently associated with septal *e*′ velocity, and GLS after adjustment for multiple cardiovascular risk factors and LVMI (Table 2).

**Table 2:** Multiple linear regression analysis between EAT thickness and myocardial function in the men and women after adjustment for age, body mass index, systolic blood pressure, diastolic blood pressure, and fasting glucose, triglyceride, high-density lipoprotein levels and LVMI

	All				Men				Women			
	β	95% CI		p	β	95% CI		p	β	95% CI		p
(a) *e*′ velocity												
EAT	− 0.163	− 0.270	− 0.057	0.003	− 0.222	− 0.363	− 0.081	0.002	− 0.048	− 0.224	0.127	0.586
Age	− 0.087	− 0.125	− 0.048	< 0.001	− 0.079	− 0.130	− 0.028	0.002	− 0.099	− 0.162	− 0.037	0.002
BMI	− 0.028	− 0.095	0.040	0.419	− 0.008	− 0.100	0.084	0.866	− 0.059	− 0.161	0.043	0.252
SBP	0.003	− 0.010	0.015	0.661	− 0.005	− 0.023	0.012	0.545	0.009	− 0.010	0.027	0.358
FBG	− 0.006	− 0.019	0.006	0.311	− 0.008	− 0.024	0.008	0.316	− 0.010	− 0.034	0.013	0.384
TG	0.001	− 0.001	0.003	0.312	0.002	− 0.001	0.004	0.221	< 0.001	− 0.004	0.004	0.917
HDL	0.013	− 0.003	0.028	0.120	0.015	− 0.010	0.039	0.238	0.017	− 0.007	0.040	0.166
LVMI	− 0.013	− 0.022	− 0.004	0.006	− 0.014	− 0.025	− 0.003	0.010	− 0.012	− 0.032	0.007	0.204
(b) GLS												
EAT	− 0.217	− 0.372	− 0.061	0.007	− 0.376	− 0.595	− 0.158	0.001	− 0.064	− 0.278	0.151	0.560
Age	0.003	− 0.054	0.060	0.916	0.019	− 0.062	0.099	0.647	− 0.004	− 0.080	0.073	0.921
BMI	− 0.077	− 0.176	0.022	0.125	− 0.041	− 0.187	0.105	0.581	− 0.101	− 0.226	0.024	0.111
SBP	− 0.020	− 0.038	− 0.002	0.030	− 0.013	− 0.041	0.015	0.352	− 0.019	− 0.041	0.004	0.101
FBG	− 0.012	− 0.030	0.006	0.199	0.013	− 0.012	0.037	0.306	− 0.028	− 0.057	0.001	0.060
TG	0.000	− 0.003	0.003	0.890	0.000	− 0.004	0.004	0.878	− 0.002	− 0.007	0.003	0.408
HDL	0.023	0.002	0.044	0.029	0.008	− 0.022	0.038	0.618	0.022	− 0.007	0.050	0.140
LVMI	− 0.024	− 0.037	− 0.010	0.001	− 0.016	− 0.033	0.001	0.070	− 0.014	− 0.038	0.010	0.241

​EAT, epicardial adipose tissue; LVMI, left ventricular mass index; *β*, standardized coefficient; CI, confidence interval; *e*′, septal early mitral tissue velocity; BMI, body mass index; SBP, systolic blood pressure; FBG, fasting blood glucose; TG, triglyceridse; HDL, high-density lipoprotein; GLS, global longitudinal strain

## Discussion

This is the first study to manifest sex-related differences in the association among EAT thickness, hs-CRP level, and myocardial function in middle-aged subjects with suspected metabolic syndrome. In the present study, we demonstrate that (1) in an asymptomatic middle aged population, components of metabolic syndrome were more impaired in men than in women; (2) EAT thickness showed no difference between men and women; (3) men had a higher LVMI and more-impaired GLS than women; (4) hs-CRP level was associated with myocardial impairment by GLS and *e*′ velocity; and (5) only men showed the significant association of EAT thickness with hs-CRP level, LVMI, and myocardial dysfunction.

### EAT and myocardial function

Although considerable studies have been conducted on the association between EAT and myocardial dysfunction, it has not been clearly elucidated [20, 22, 27]. In the present study, EAT correlated with LVMI, *e*′, and GLS. Our findings collaborate to involve EAT with the pathogenic mechanism of myocardial remodeling and subclinical myocardial dysfunction in middle-aged subjects without cardiovascular disease [22, 28]. As EAT thickness is thought to reflect the amount of VAT, the effect of EAT on atherosclerotic cardiovascular disease is considered a consequence of enhanced insulin resistance and abnormal lipid metabolism. However, various pathogenic mechanisms have been suggested about the influence of EAT on myocardial function. First, because accumulated EAT envelopes the right ventricular free wall, the paracrine effects of EAT-derived adipocytokines and inflammatory mediators could directly influence the myocardium. Second, these cytokines could develop coronary atherosclerosis and then damage the myocardium through myocardial ischemia. In a study with multi-detector computed tomography coronary angiography, EAT thickness surrounding the left anterior descending artery predicted the presence, extent and severity of coronary artery disease after adjustment for confounding factors [29]. Furthermore, in patients with acute coronary syndrome, perivascular fat stranding was related to the culprit lesion, a lower Agatston score, presence of regional wall motion abnormalities, and initial elevation of serum troponin levels [30]. Our cohort study is an ongoing prospective study, and follow-up data of coronary events, including myocardial infarction and any coronary revascularization, will be investigated. The direct mechanical effect of thick EAT could limit and compress myocardial motion [20, 31]. Our previous study that indicated that lateral *e*′ and *s*′ (systolic mitral annular velocity) have a stronger relationship than septal *e*′ and *s*′ velocity supports this hypothesis [21]. Left atrial (LA) dysfunction has also been suggested as a possible mechanism to link EAT and LV diastolic dysfunction. Evin et al. reported that magnetic resonance imaging derived LA strain correlates well with BMI and EAT volume in patients with type 2 diabetes and obesity, suggesting that LA strain could be a sensitive tool for detection of early LV diastolic dysfunction [32].

### EAT and secretome profile

EAT is considered as an endocrine organ of bioactive molecules, including various inflammatory mediators. The association between EAT and several adipocytokines has been suggested. Secreted frizzled-related protein 4, a novel adipokine which is related to insulin resistance was increased in human EAT samples in patients with coronary artery disease [33]. EAT derived omentin-1, another novel adipokine which inhibits inflammation and improves insulin resistance was reduced in patients with coronary artery disease [34]. In patients with coronary artery disease, human samples of EAT obtained during elective cardiac surgery demonstrated enhanced inflammatory cell infiltration and inflammatory cytokine activity [14]. This locally enhanced inflammation may affect the pathophysiology of coronary atherosclerosis. Adipose tissue is a source of hs-CRP production that has a reciprocal association with adiponectin and anti-inflammatory protein levels [35]. Previous studies reported that EAT is significantly associated with hs-CRP level as a marker of low-grade systemic inflammation [13, 36]. However, to our knowledge, only few studies investigated the association among EAT, hs-CRP level, and myocardial function. One small-sample study with hypertensive patients reported that increased EAT and hs-CRP level are related with LV diastolic dysfunction [37]. In our study, increased EAT thickness and hs-CRP level were closely associated with subclinical myocardial dysfunction, including *e*′ and GLS as LV diastolic and systolic functions, respectively.

### EAT and metabolic profile

Among other visceral adipose tissues, EAT has the greatest capacity for free fatty acid (FFA) release and uptake. In normal physiology, this FFA provides energy for the myocardium. This mechanism of EAT buffers the myocardium from exposure of high FFA concentration [19]. However, in pathological conditions, the intense metabolic activity of EAT could contribute to myocardial damage. In previous studies, EAT was related to myocardial TG content, LV concentric remodeling, and myocardial dysfunction [38, 39]. This suggests that myocardial infiltration of TG could induce direct myocardial damage. The present study demonstrates that EAT correlates with TG level and men have higher TG levels and TG/HDL ratios than women. Our finding implicates that the high metabolic activity of TG in middle-aged men may induce myocardial TG accumulation and myocardial remodeling and dysfunction.

### Sex-related differences

Our study with a middle-aged general population demonstrates that only men had a significant association between EAT thickness and myocardial dysfunction, although EAT thickness was not different each other. Currently, the sex difference in the association between EAT and myocardial dysfunction has not been clearly investigated, but a possible cause could be sexual dimorphism of EAT during aging. In female aged rats, the level of EAT genes for interleukin-6 (IL-6), plasminogen activator inhibitor-1 (PAI-1), adiponectin and peroxisome proliferators activated receptor γ were lower than in young rats. By comparison, aged male rats showed an increased expression of genes for inflammatory cytokine, IL-6, in EAT [40]. Changes in obesity related genes in EAT were observed only in female rats, but not in males during aging [41]. This indicates that a dysfunctional EAT associated with aging demonstrates different metabolic and inflammatory activities between the two sexes. The sex difference of EAT function might be responsible for the sex difference of EAT-related inflammation and myocardial dysfunction.

Estrogen has a protective role in obesity induced chronic inflammation. Estrogen has immunosuppressive activity, as demonstrated by the effect of 17- β estradiol suppressing proinflammatory T helper 17 cells and M1 macrophage cell differentiation. Moreover, estrogen receptors are expressed on the surface of immune cells and modulate immune activity [42, 43]. Estrogen is also a determining factor for metabolism and function of adipose tissue. In mice models, estrogen prevented impaired glucose tolerance and obesity by regulating adipogenesis, lipolysis, and lipogenesis [44]. Ovariectomy of female rats decreased the vasodilatory effects of perivascular adipose tissue compared to sham rats [45]. Menopause has been reported to occur at about 50 years of age. Thus, majority of the women in this study may have post-menopausal hypoestrogenism. However, the prevalence of CVD rapidly increases after 5–10 years after menopause [46]. Although the age of menopause is not exactly consistent with the mean age of the study population, the protective effect of estrogen over the preceding 50 years could be considered as a factor that explains the sex difference in myocardial function in middle aged population.

Recent studies have reported that EAT has the characteristic of brown adipose tissue. Sacks et al. demonstrated that EAT has high expression of mitochondrial uncoupling protein-1 which is the main marker of brown adipose tissue [47]. Estrogen could increase this gene expression [48] and influence mitocondriogenesis via estrogen receptor-α of the mitochondria of brown adipose tissue [49]. Therefore the brown adipose tissue characteristic of EAT, which was associated with a more favorable risk of cardiovascular disease might be relatively small in men than women [50, 51].

Recently, in a study involving 56 healthy women, increased EAT thickness was significantly associated with increased thrombotic risk, assessed by PAI-1 [52]. To explain the sex difference, the association between EAT and thrombotic activity in men and women should be investigated and discussed in future studies.

### Clinical implications

Our study enrolled an asymptomatic middle-aged population without cardiovascular diseases but with suspected metabolic syndrome. Therefore, our finding suggests that EAT thickness could be a marker of the early processes of cardiac remodeling and dysfunction in these subjects. Furthermore, the present study demonstrates that EAT and hs-CRP level as a proinflammatory marker is closely associated with subclinical myocardial function in middle-aged men. Combined with the increasing evidence of the association between inflammation and cardiovascular disease [53], our findings might be a basis for applying medications with anti-inflammatory effects in this middle-aged population.

### Study limitations

This study has several limitations. We included in the analysis subjects who agreed to undergo additional speckle tracking study, which can detect subtle changes of myocardial function even in asymptomatic people. However, demographic data, including age, sex, BMI, waist circumference, and blood pressure, did not significantly differ between our 277 subjects and the overall cohort. Second, the cross-sectional analysis of this study could not prove a clear causality with EAT. Our ongoing follow-up study may provide the answer. Third, although mean age and BMI were not significantly different, men had more-impaired metabolic parameters, including blood pressure, and FBG, TG, and adiponectin levels. However, these findings are consistent with those of previous large cohort studies [44, 45]. The sex-related difference in the metabolic component in our study could be representative of that in the real-world population. Moreover, after the multiple linear regression analysis that adjusted for traditional cardiovascular risk factors, EAT remained a significant independent factor of myocardial remodeling and subclinical dysfunction. However, the effects of residual confounding factors cannot be excluded. Lastly, EAT is a well-known source of adipocytokines and inflammatory mediators, but this study only demonstrates the association between EAT and hs-CRP level as a low-grade inflammation marker.

## Conclusion

In middle-aged subjects with suspected metabolic syndrome, EAT was associated with inflammation represented by hs-CRP level, LV mass, and subclinical myocardial dysfunction only in men. These findings may suggest that the inflammatory activity of EAT induced myocardial remodeling and dysfunction in the middle-aged subjects and was attenuated in the women.

## Abbreviations

CVD: cardiovascular diseases
BMI: body mass index
VAT: visceral adipose tissue
EAT: epicardial adipose tissue
2-D: two-dimensional
SBP: systolic blood pressure
DBP: diastolic blood pressure
FBG: fasting blood glucose
TC: total cholesterol
LDL: low-density lipoprotein
HDL: high-density lipoprotein
hs-CRP: high-sensitivity C-reactive protein
LA: left atrial
LAVI: left atrial volume index
LV: left ventricular
LVMI: left ventricular mass index
E + A: velocity of early filling and late filling
*e*′: early diastolic mitral annular velocity
GLS: global longitudinal strain
CI: confidence interval
*s*′: systolic mitral annular velocity
FFA: free fatty acid
IL-6: interleukin-6
PAI-1: plasminogen activator inhibitor-1
